# Risk of Iatrogenic Peroneal Nerve Injury in Inside-Out Lateral Meniscal Repairs Using Differently Curved Repair Devices and Surgical Portals

**DOI:** 10.3390/jcm14062007

**Published:** 2025-03-16

**Authors:** Wachiraphan Parinyakhup, Tanarat Boonriong, Prapakorn Klabklay, Korakot Maliwankul, Hafizz Sanitsakul, Chaiwat Chuaychoosakoon

**Affiliations:** Department of Orthopedics, Faculty of Medicine, Prince of Songkla University, Hat Yai, Songkhla 90110, Thailand; wachirapun.p@psu.ac.th (W.P.); tanarat.b@psu.ac.th (T.B.); prapagorn.g@psu.ac.th (P.K.); drkorakot.m@gmail.com (K.M.); fizzortho@gmail.com (H.S.)

**Keywords:** peroneal nerve injury, meniscal repair, lateral meniscus, repair device curvature, surgical portals, MRI analysis, safe zones, iatrogenic complications

## Abstract

**Background:** Inside-out meniscal repair is a widely adopted treatment for lateral meniscal injuries. A significant complication associated with this procedure is iatrogenic peroneal nerve (PN) injury, reported in approximately 9% of cases. The risk varies depending on the choice of surgical portals, curvature of repair devices, and anatomical landmarks. This study aimed to assess the risk of PN injury and define safe zones for inside-out lateral meniscal repair using different device curvatures and portal combinations. **Methods:** Axial MRI scans of knees positioned in the figure-of-four posture, with joint fluid distension and varus force applied, were analyzed in 29 adult patients. Transparent overlays representing the operative routes of the anterior-, middle-, and posterior-curved needles were superimposed on the MRI scans. Simulations of repair procedures were performed using the anteromedial, accessory anteromedial, anterolateral, and accessory anterolateral portals, targeting the medial and lateral borders of the popliteus tendon (PT). Instances where the needle path intersected or contacted the PN were recorded to delineate risk zones. **Results:** Repairs targeting the medial PT border with anterior-curved devices via the anteromedial or accessory anteromedial portals were identified as safe. At the lateral PT border, all device curvatures and portals were considered safe, except for middle- and posterior-curved devices used through the accessory anteromedial portal, which posed a risk of PN injury. **Conclusions:** The risk of iatrogenic PN injury in inside-out lateral meniscal repair depends on the curvature of the repair device and portal used. Adhering to the identified safe zones can substantially reduce this risk.

## 1. Introduction

Lateral meniscal tears are common knee injuries observed across various age groups [1,2], particularly in physically active individuals and those with anterior cruciate ligament (ACL) injuries [3]. The incidence ranges from 0.3 to 1.2 per 1000 person-years in the general population, with a male-to-female ratio of approximately 2:1 [4], likely due to higher participation in high-impact sports and occupational activities. While magnetic resonance imaging (MRI) is a highly accurate and noninvasive method for detecting meniscal injuries, studies suggest that a thorough clinical examination by an experienced physician can provide comparable diagnostic accuracy, reducing unnecessary imaging [5,6,7,8]. Although isolated lateral meniscal tears are less frequent than medial meniscal tears, they are more commonly observed in individuals under 20 years of age, whereas medial tears become more prevalent with aging [9]. Over the past decades, the incidence of meniscal repair procedures has significantly increased, with a 320% rise in Europe and a twofold increase in the United States between 2005 and 2011 [10]. Lateral meniscal injuries not only cause pain and mechanical symptoms but also impair knee mobility and stability, significantly affecting activities of daily living, physical functioning, and quality of life. If left untreated or improperly managed, these injuries contribute to progressive osteoarthritis, increasing years lived with disability and imposing a substantial economic burden through rising healthcare costs, rehabilitation expenses, and lost productivity [11,12]. As the understanding of the crucial role of the lateral meniscus in knee biomechanics has evolved, treatment strategies have shifted toward meniscal preservation rather than meniscectomy to mitigate long-term adverse outcomes.

Lateral meniscal repair is recommended for patients with meniscal injury, as early intervention reduces the risk of osteoarthritis development [13]. Surgeons can perform this repair using the all-inside, inside-out, or outside-in techniques. All-inside meniscal repair [14] is popular due to its ease of performance and time efficiency; however, it has a high failure rate because of the thinness of the lateral joint capsule [13]. Conversely, the inside-out technique has demonstrated superior outcomes, with a significantly lower failure rate than the all-inside lateral meniscal repair technique [15]. Additionally, it effectively reduces meniscal extrusion and posterior shifts in lateral meniscal tears. When combined with ACL reconstruction, the success rate for inside-out meniscal repairs can reach up to 90% [16,17], likely due to a more conducive healing environment. However, this technique carries a risk of iatrogenic peroneal nerve injury [18,19,20], as the peroneal nerve is located near the popliteus tendon and knee joint capsule [21]. A systematic review reported an incidence of nerve irritation or injury up to 9% [22].

MRI [23] and cadaver [24] studies have evaluated the risk of iatrogenic peroneal nerve injury during inside-out lateral meniscal repair; however, these studies had specific limitations. MRI evaluations were conducted with knees positioned at 30° flexion using standard MRI techniques, without varus force or joint fluid dilatation, conditions that do not replicate those during actual arthroscopic lateral meniscal repair. Conversely, cadaveric studies used knee-section cadavers that lacked soft tissue tension and could not simulate the figure-of-four position with applied varus force. While MRI studies lacked realistic knee positions and joint fluid dilatation, cadaveric studies lacked soft tissue tension, making them unsuitable for assessing risk in clinically relevant positions. No prior studies have evaluated risk in positions replicating arthroscopic lateral meniscal repair.

Thus, in this study, we evaluated the risk of iatrogenic peroneal nerve injury using MRI sections of the knee in the arthroscopic lateral meniscal repair position (figure-of-four position with varus force and joint fluid dilatation). Different curved inside-out meniscal repair devices were deployed from various portals, and their relation to the medial and lateral borders of the popliteus tendon was assessed. We hypothesized that the inside-out lateral meniscal repair technique presents a risk to the peroneal nerve, with the degree of risk varying based on the type of curved device and portal used.

## 2. Materials and Methods

This prospective study was conducted in accordance with the Declaration of Helsinki and approved by the Institutional Review Board (or Ethics Committee) of the Faculty of Medicine of Prince of Songkla University (REC 60-180-11-1 and 66-456-11-1). It included 79 patients who underwent arthroscopic ACL reconstruction between 1 January 2018 and 31 December 2020. A total of 50 patients were excluded due to the presence of medial or lateral meniscal ligament injuries, concurrent meniscal and/or cartilage procedures, or a prior history of knee surgery.

**Figure 1:**
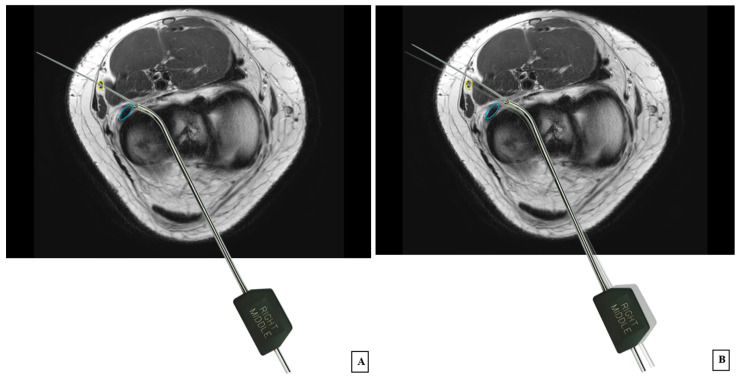
Axial MRI image of the knee showing (**A**) the needle passing through or touching the peroneal nerve, indicating a risk of iatrogenic peroneal nerve injury; (**B**) a second transparent sheet printed with the same curve as the zone-specific device was placed from the same portal to the outer border of the peroneal nerve. The “danger zone” was defined as the area between the two zone-specific devices at the level of the meniscocapsular junction. (Blue circle: popliteus tendon; yellow circle: peroneal nerve).

After each operation, patients were immediately taken for postoperative MRI of the knee. Before transferring the patients to the MRI room, joint fluid pressure was increased to 50 mmHg using an arthroscopic pump system to maintain knee joint distention. Using axial MRI images obtained with the knee in the figure-of-four position and joint fluid dilatation, combined with varus force at the level of the lateral meniscus, transparent sheets printed with combined zone-specific devices and anterior, middle, and posterior curved needles were used to evaluate the risk of iatrogenic peroneal nerve injury and identify safe and danger zones in relation to the medial and lateral borders of the popliteus tendon. The anteromedial and accessory anteromedial portals were positioned at the medial border and 5 mm from the medial border of the patellar tendon, respectively. Similarly, the anterolateral and accessory anterolateral portals were positioned at the lateral border and 5 mm from the lateral border of the patellar tendon. Transparent sheets printed with zone-specific devices were placed over the axial MRI images to trace the needle trajectory from the anteromedial, accessory anteromedial, anterolateral, or accessory anterolateral portal to the medial or lateral border of the popliteus tendon. The closest distance between the needle and the peroneal nerve border was measured. If the overlaid images showed the needle passing through or touching the peroneal nerve (Figure 1A), the risk of iatrogenic peroneal nerve injury was recorded. A second transparent sheet, printed with the same curve as the zone-specific device, was placed from the same portal to the outer border of the peroneal nerve (Figure 1B). The “danger zone” was defined as the area between the two zone-specific devices at the level of the meniscocapsular junction (Figure 1B).

All measurements were taken three times by an experienced musculoskeletal radiologist. Interobserver reliability was assessed using kappa statistics for categorical variables and intraclass correlation coefficients for continuous variables. Findings are presented as descriptive statistics (means ± SDs) to indicate the risk of iatrogenic peroneal nerve injury. Statistical analyses were performed using the R program with the Epicalc package (version 3.4.3; R Foundation for Statistical Computing, Vienna, Austria). Associations and the statistical significance of the observed risks were assessed using chi-square tests, with a *p*-value of <0.05 considered statistically significant. Continuous variables were compared using the Wilcoxon rank-sum test, with Bonferroni correction applied to adjust for multiple comparisons. For categorical variables with more than two levels, pairwise comparisons were conducted using the Pairwise Nominal Independence Test with Fisher’s exact test, also adjusted for multiple comparisons using Bonferroni correction. This approach ensured a rigorous statistical analysis, minimizing the risk of Type I errors while maintaining appropriate sensitivity for detecting significant differences.

The determination of the necessary sample size for comparing the mean distances from the needle tip to the peroneal nerve via the anteromedial and anterolateral portals was informed by a prior study [25], which suggested a minimum requirement of 17 MRI images. To ensure sufficient statistical power, this sample size was re-evaluated using data from an alternative study [26], which recommended a sample size of 29. Consequently, a larger sample size was adopted for the analysis.

## 3. Results

A total of 29 knee MRI scans (26 from male patients and 3 from female patients) were included in the study. The patients had a mean age of 31.4 ± 10.7 years, with an average weight of 68.66 ± 8.11 kg and height of 170.07 ± 4.52 cm. Of these 29 MRI scans, two were from knees that had undergone surgery to remove a loose body and 27 were from knees that had undergone ACL reconstruction.

We observed no significant risk when repairing the lateral meniscal tissue using anterior-, middle-, or posterior-curved zone-specific devices through the anteromedial, accessory anteromedial, or anterolateral portals in relation to the lateral border of the popliteus tendon. Similarly, no significant risk was found when using the anterior-curved zone-specific device through the accessory anterolateral portal in relation to the lateral border of the popliteus tendon. However, the risks of iatrogenic injury with repair through the accessory anterolateral portal using middle- and posterior-curved devices were 6.89% and 10.34%, respectively. The risks associated with various repair device options for the medial border of the popliteus tendon are shown in Table 1, where a significant risk was noted only when using the middle-curved zone-specific device through the anteromedial portal compared to the anterior-curved device. The average and maximum danger zones in repairing the lateral meniscal tissue using anterior-, middle-, or posterior-curved zone-specific devices through the anteromedial, accessory anteromedial, and anterolateral portals in relation to the medial and lateral borders of the popliteus tendon are shown in Table 2. Interobserver reliability for all measurements exceeded 0.9 (Table 3), demonstrating high measurement consistency.

## 4. Discussion

In this study, we evaluated the risk of iatrogenic peroneal nerve and posterior neurovascular injuries using an inside-out technique with MRIs of the knee in the arthroscopic lateral meniscal repair position and using differently curved repair devices deployed through different portals. We found no risk of peroneal nerve injury when repairing through the anteromedial, accessory anteromedial, anterolateral, and accessory anterolateral portals in relation to the lateral borders of the popliteus tendon with all three curved devices. However, repairs through the accessory anterolateral portal with the middle- and posterior-curved devices showed incidences of 6.9% and 10.34%, respectively. Regarding the medial border of the popliteus tendon, the risks of iatrogenic peroneal nerve injury when repairing through the anteromedial, accessory anteromedial, anterolateral, and accessory anterolateral portals were 0%, 0%, 20.69%, and 13.79%, respectively, with the anterior-curved device; 24.14%, 20.69%, 20.69% and 17.24%, respectively, with the middle-curved device; and 17.24%, 13.79%, 20.69%, and 13.79%, respectively, with the posterior-curved device.

This study provides a clinically relevant assessment of iatrogenic peroneal nerve injury risk during inside-out lateral meniscal repair. Unlike previous cadaveric and MRI-based studies, it incorporates MRI under arthroscopic conditions that closely mimic actual surgical procedures. Key factors such as figure-of-four positioning, varus force application, and joint fluid distension were considered, improving the accuracy of risk evaluation. The use of differently curved repair devices and multiple portal combinations further refines the identification of high-risk and safe zones. By bridging the gap between anatomical research and clinical practice, this study offers practical guidelines for optimizing portal selection and device choice, ultimately enhancing patient safety and surgical outcomes.

Lateral meniscal repair poses a risk of iatrogenic peroneal nerve injury due to the proximity of the nerve to the popliteus tendon and joint capsule. An earlier study associated the use of an all-inside meniscal repair device in this area with a high failure rate (19%) [27], which was attributed to the thinness of the joint capsule. To minimize this risk, inside-out lateral meniscal repair is recommended, although it still poses a potential risk to the peroneal nerve. Various reports of complications following inside-out repair have documented this risk. For instance, Laprade et al. described a case of peroneal nerve injury after arthroscopic inside-out lateral meniscal repair, which was resolved with late neurolysis [28]. In another study by Rizzo et al., a patient undergoing inside-out repair for a complex radial tear of the posterior horn and body of the lateral meniscus experienced postoperative decreased sensation in the right lower leg and weakness in ankle dorsiflexion and great toe extension. Despite initial management, these symptoms persisted for two weeks. Further diagnostics, including an MRI, revealed an enlarged and inflamed right common peroneal nerve. Surgical exploration revealed a suture encircling the nerve, which was removed to alleviate nerve entrapment and associated symptoms. Additionally, Bruzzone et al. reported that performing an inside-out release technique on the posterolateral corner during total knee arthroplasty poses a risk of peroneal nerve injury due to its proximity to the nerve [29].

MRI-based and cadaveric studies have been conducted to reduce the risk of peroneal nerve injury during inside-out lateral meniscal repair. Gupta et al. assessed the risk of neurovascular injury in inside-out meniscal repairs using standard knee MRIs with 30° knee flexion [23]. The study used the center of the tibial plateau as the reference point for clock positions to simplify reporting. However, applying these clock positions to real operative situations is challenging, as their measurements do not reflect the standard arthroscopic setup. Additionally, the risk may differ in actual surgeries because (1) the center of rotation of the meniscal repair device is at the portal and (2) the knee position in their study differed from the standard lateral meniscal repair position (figure-of-four with varus force and joint fluid dilation). In a cadaveric study, Atbaşı et al. assessed the risk of peroneal nerve injury at full knee extension and with 90° knee flexion using a mid-leg to mid-thigh knee section [24]. K-wires were inserted into the anterior, middle, and posterior horns of the lateral meniscus and the distances from the wire tips to the peroneal nerve were measured. This revealed that repairs at the posterior horn were safe. However, the study had two significant limitations. First, cadaveric knee sections have different soft tissue tension compared to live patients. Second, the straight K-wires used did not accurately represent the curved shapes of standard inside-out meniscal repair devices.

This study had some limitations. First, the accessory portals were set 5 mm from the border of the patellar tendon, so surgeons should use the results cautiously when creating portals more or less than 5 mm from the tendon. Second, the risk was evaluated by simulating device paths parallel to the joint line. If the surgeon rotates the device during repair, the risk may differ from our findings. Third, we used the popliteus tendon as the reference landmark, which passes through the knee joint in an oblique direction and might differ when using different axial MRI images. We minimized this potential issue by selecting axial MRI images passing through the center of the lateral meniscus. Additionally, a key limitation of this study is the relatively small sample size (N = 29), which may affect the generalizability of the findings. Although the methodology relies on precise MRI analyses, anatomical variations within the general population may not have been fully captured. To enhance the reliability of future studies, expanding the analysis to a larger cohort and incorporating data from multiple clinical centers is recommended. A larger sample size would allow for a more comprehensive assessment of the “danger zones” and their clinical implications.

For future research directions, studies on inside-out lateral meniscal repair should address current limitations and incorporate key advancements to enhance accuracy, clinical relevance, and applicability. First, larger sample sizes with diverse patient demographics should be included to account for anatomical variations in the popliteus tendon and peroneal nerve trajectory, which may affect the risk of nerve injury. Second, future research should utilize three-dimensional MRI reconstructions or dynamic imaging to provide a more precise assessment of needle trajectory and nerve proximity in different knee positions. Third, comparative studies evaluating different surgical techniques, such as all-inside, inside-out, and outside-in approaches, should be conducted to identify the safest and most effective method for lateral meniscal repair.

## 5. Conclusions

Based on our findings, lateral meniscus repairs are safe when performed through anteromedial, accessory anteromedial, or anterolateral portals using anterior-, middle-, or posterior-curved zone-specific devices relative to the lateral border of the popliteus tendon. Similarly, repairs using the anteromedial and accessory anteromedial portals with an anterior-curved device are safe, relative to the medial border of the popliteus tendon. However, inside-out repairs performed in relation to the medial border endangered the peroneal nerve with certain device and portal combinations. Overall, all repairs were safe when executed relative to the lateral border of the popliteus tendon. Surgeons can use these identified safe zones to reduce the risk of accidental peroneal nerve damage during lateral meniscal repairs.

## Figures and Tables

**Table 1:** Risks of iatrogenic peroneal nerve injury in simulated lateral meniscal repair using the inside-out technique in relation to the medial and lateral borders of the popliteus tendon, with differently curved needles deployed from various portals. (AM: anteromedial, aAM: accessory anteromedial, AL: anterolateral, aAL: accessory anterolateral, PT: popliteus tendon).

Reference	Portal	Curve (%)			*p*-Value A-M	*p*-Value A-P	*p*-Value M-P
		Anterior (A)	Middle (M)	Posterior (P)			
Medial border of PT	AM	0.00	24.14	17.24	0.03 *	0.16	1.00
	aAM	0.00	20.69	13.79	0.07	0.34	1.00
	AL	20.69	20.69	20.69	1.00	1.00	1.00
	aAL	13.79	17.24	13.79	0.72	1.00	0.72
Lateral border of PT	AM	0.00	0.00	0.00	-	-	-
	aAM	0.00	0.00	0.00	-	-	-
	AL	0.00	0.00	0.00	-	-	-
	aAL	0.00	6.90	10.34	1.00	0.711	1.00

* Statistically significant.

**Table 2:** “Danger zones” in repairing lateral meniscal tissue using anterior-, middle-, or posterior-curved zone-specific devices through the anteromedial, accessory anteromedial, and anterolateral portals in relation to the medial and lateral borders of the popliteus tendon. (AM: anteromedial, aAM: accessory anteromedial, AL: anterolateral, aAL: accessory anterolateral, PT: popliteus tendon).

Reference	Portal	Curve (Mean ± SD)			*p*-Value A-M	*p*-Value A-P	*p*-Value M-P
		Anterior (A)	Middle (M)	Posterior (P)			
Medial border of PT	AM	-	3.62 ± 1.00	3.15 ± 1.13	-	-	0.53
	aAM	-	3.94 ± 1.58	3.65 ± 0.85	-	-	0.91
	AL	4.66 ± 2.80	4.34 ± 1.07	3.92 ± 1.13	1.00	1.00	1.00
	aAL	4.69 ± 1.66	2.74 ± 1.39	3.71 ± 1.40	0.33	1.00	0.86
Lateral border of PT	AM	-	-	-	-	-	-
	aAM	-	-	-	-	-	-
	AL	-	-	-	-	-	-
	aAL	-	2.10 ± 0.54	1.66 ± 0.34	-	-	0.40

**Table 3:** Interobserver reliability scores for all measurements.

Reference	Portal	Curve (ICC (95%CI))		
		Anterior (A)	Middle (M)	Posterior (P)
Medial border of PT	AM	0.997 (0.995–0.999)	0.998 (0.996–0.999)	0.999 (0.998–0.999)
	aAM	0.995 (0.991–0.998)	0.998 (0.997–0.999)	0.999 (0.998–0.999)
	AL	0.994 (0.989–0.997)	0.996 (0.993–0.998)	0.997 (0.995–0.999)
	aAL	0.994 (0.988–0.997)	0.997 (0.995–0.999)	0.998 (0.996–0.999)
Lateral border of PT	AM	0.997 (0.994–0.998)	0.999 (0.997–0.999)	0.999 (0.998–0.999)
	aAM	0.996 (0.992–0.998)	0.999 (0.997–0.999)	0.999 (0.997–0.999)
	AL	0.998 (0.996–0.999)	0.998 (0.997–0.999)	0.991 (0.984–0.996)
	aAL	0.997 (0.995–0.999)	0.999 (0.998–0.999)	0.999 (0.997–0.999)

## Data Availability

Data supporting the findings are available upon request from the corresponding author.

## Abbreviations

The following abbreviations are used in this manuscript:

ACL	Anterior cruciate ligament
MRI	Magnetic resonance imaging
AM	Anteromedial portal
AL	Anterolateral portal
aAM	Accessory anteromedial portal
aAL	Accessory anterolateral portal
PT	Popliteus tendon
